# Crystal Structure and Substrate Specificity of *Drosophila* 3,4-Dihydroxyphenylalanine Decarboxylase

**DOI:** 10.1371/journal.pone.0008826

**Published:** 2010-01-21

**Authors:** Qian Han, Haizhen Ding, Howard Robinson, Bruce M. Christensen, Jianyong Li

**Affiliations:** 1 Department of Biochemistry, Virginia Tech, Blacksburg, Virginia, United States of America; 2 Biology Department, Brookhaven National Laboratory, Upton, New York, United States of America; 3 Department of Pathobiological Sciences, University of Wisconsin, Madison, Wisconsin, United States of America; Griffith University, Australia

## Abstract

**Background:**

3,4-Dihydroxyphenylalanine decarboxylase (DDC), also known as aromatic L-amino acid decarboxylase, catalyzes the decarboxylation of a number of aromatic L-amino acids. Physiologically, DDC is responsible for the production of dopamine and serotonin through the decarboxylation of 3,4-dihydroxyphenylalanine and 5-hydroxytryptophan, respectively. In insects, both dopamine and serotonin serve as classical neurotransmitters, neuromodulators, or neurohormones, and dopamine is also involved in insect cuticle formation, eggshell hardening, and immune responses.

**Principal Findings:**

In this study, we expressed a typical DDC enzyme from *Drosophila melanogaster*, critically analyzed its substrate specificity and biochemical properties, determined its crystal structure at 1.75 Angstrom resolution, and evaluated the roles residues T82 and H192 play in substrate binding and enzyme catalysis through site-directed mutagenesis of the enzyme. Our results establish that this DDC functions exclusively on the production of dopamine and serotonin, with no activity to tyrosine or tryptophan and catalyzes the formation of serotonin more efficiently than dopamine.

**Conclusions:**

The crystal structure of *Drosophila* DDC and the site-directed mutagenesis study of the enzyme demonstrate that T82 is involved in substrate binding and that H192 is used not only for substrate interaction, but for cofactor binding of drDDC as well. Through comparative analysis, the results also provide insight into the structure-function relationship of other insect DDC-like proteins.

## Introduction

3,4-Dihydroxyphenylalanine (DOPA) decarboxylase (DDC) is a pyridoxal-5′-phophate (PLP)-dependent enzyme which physiologically catalyzes the decarboxylation of 3,4-dihydroxyphenylalanine to dopamine, and 5-hydroxytryptophan (5-HTP) to 5-hydroxytryptamine (serotonin) [1], [2]. Because the enzyme catalyzes the decarboxylation of other aromatic L-amino acids as well, it is commonly named aromatic L-amino acid decarboxylase. The enzyme is present in the majority of living species from select bacteria to humans. DDC from mammals and insects has attracted significant attention because both dopamine and serotonin serve as classical neurotransmitters, neuromodulators, or neurohormones [3], [4], [5], [6]. A DDC inhibitor is being used in conjunction with DOPA in humans for the treatment of Parkinson's disease [7]. In insects DDC occupies a special position because dopamine is a key precursor in insect cuticle formation [8], [9], eggshell hardening [10], [11] and is involved in immune responses [8], [12], [13], [14], [15], [16], [17].

A protein BLAST (Basic Local Alignment Search Tool) search using pig DDC sequence shows that *Drosophila* has at least five aromatic L-amino acid decarboxylases or DDC-like sequences, but mammals have only one. In *Drosophila*, one of the five DDC-like sequences is the typical DDC, two of the DDC-like sequences have been proposed to be tyrosine decarboxylases, and the other two DDC-like sequences have been named α-methyl DOPA-resistant proteins. The role of tyrosine decarboxylase was proposed for two of the DDC-like sequences based on the negative effect on tyramine production of their mutations [18], [19], [20], [21], [22], but the biochemical characteristics of these proposed tyrosine decarboxylases have not been clearly established. The α-methyl DOPA-resistant protein was named based on the observation that *Drosophila* having this protein were resistant to the toxic compound α-methyl DOPA [23], [24], [25], [26]. The insect DDC-like sequences share high sequence identity with one another (>40%) and their level of identity increases to ∼50% when only their putative catalytic domains are compared. The presence of multiple DDC-like sequences in insects raises an important question – why have multiple DDC-like sequences evolved in insects and what are their structure-function relationships? In this study, we determined the crystal structure of the typical *Drosophila* DDC (drDDC). Based on what we found in the structure, we further performed site-directed mutagenesis of the enzyme, tested the substrate specificity and identified residues that affect the substrate binding affinity and the turnover number of the enzyme. Our results provide insight into substrate specificity for drDDC.

## Results

### Biophysical Properties of drDDC Wild-Type (WT) and Mutant Proteins

**Figure 1:**
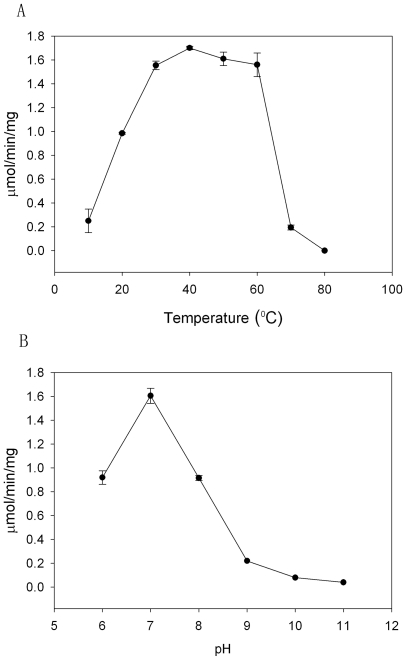
Effect of temperature and pH on drDDC activity. The activities of recombinant drDDC at different temperatures (A) and at different pH values (B).

Three site directed mutations, T82A, T82S and H192W, as well as the wildtype drDDC were investigated. Based on SDS-PAGE analysis, all four recombinant proteins were of high purity (data not shown). Freezing and thawing did not result in activity changes of the isolated enzymes. drDDC WT protein was tested for optimal temperature and pH. It showed maximum activity at 30 to 60°C (Fig. 1a), and at pH 7 (Fig. 1b).

### Overall Structure

**Figure 2:**
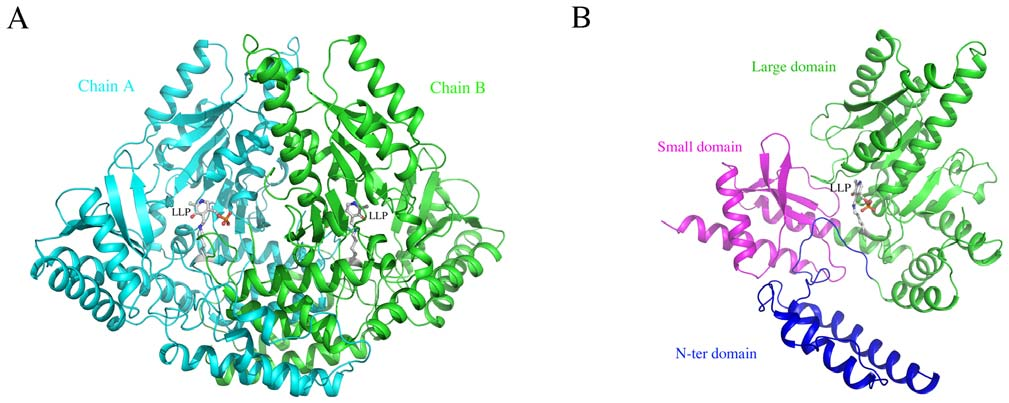
Overall structure and schematic view of one subunit of drDDC. *A,* A schematic representation of the structure of drDDC dimer. *B,* The schematic view of a monomer. The cofactor (LLP) is included in stick. Three parts, large domain (green), small domain (blue) and N-terminal part (pink) are labeled.

The structure of drDDC was determined by molecular replacement and refined to 1.75 Å resolution with excellent statistics (Table 1). The final model contains 2×448 amino acid residues and yields a crystallographic R value of 19.7% and an R_free_ value of 22.6%. All residues in the two subunits are in allowed regions of the Ramachandran plot, as defined with PROCHECK [27]. Table 1 illustrates refinement statistics of drDDC and the statistics on Ramachandran plot. There are two protein molecules in an asymmetric unit, which forms a biological homodimer. The fragment of V322 to P348 of both subunits in the structure was highly disordered; therefore, they were not included in the final drDDC model. An overview of the dimeric structure model is illustrated in Fig. 2a. Each of the two monomers is composed of three distinct domains (Fig. 2b). The large domain (Residues 86–380) contains a seven-stranded beta sheet, and the small domain comprises the C-terminal part of the chain (residues 380–475), which folds into a 4-stranded beta sheet covered with helices on one side. Based on the above features of the structure, drDDC is a fold-type I PLP-dependent enzyme [28], [29], [30], [31], [32]. In addition to these two domains, drDDC contains an additional N-terminal domain (residues 1–85). This domain is composed of two parallel helices and one small helix. This structure lies like a flap over the top of the second subunit and *vice versa*, with the first helix of one subunit aligning antiparallel to the equivalent helix of the other subunit. The drDDC architecture represents the same sub-fold type as that of pig DDC [33].

**Table 1:** Data collection and refinement statistics of drDDC.

Crystal Data	
Space Group	P2_1_2_1_2
Unit Cell	
a (Å)	105.8
b (Å)	108.6
c (Å)	86.3
α = β = γ (°)	90.0
Data collection	
X-ray source	BNL^a^-X29
Wavelength (Å)	1.0809
Resolution (Å) b	1.75 (1.81–1.75)
Total number of reflections	1508, 676
Number of unique reflections	100, 700
R-merge b	0.06 (0.56)
Redundancy b	15.1 (11.8)
Completeness (%)^b^	98.9 (97.8)
Refinement statistics	
R-work (%)	19.7
R-free (%)	22.6
RMS Bond lengths (Å)	0.014
RMS Bond angles (°)	1.423
No. of ligand or cofactor molecules	2 LLP c
	1 GOL^c^
No. of water molecules	601
Average B overall (Å^2^)	30.0
RMSD of B-factors for main chain residues (Å)	0.904
RMSD of B-factors for side chain residues (Å)	2.444
Statistics on Ramachandran plot (%)	
Most favoured regions	91.6
Additional allowed regions	8.1
Generously allowed regions	0.3

a Brookhaven National Laboratory.

b The values in parentheses are for the highest resolution shell.

c LLP, lysine-pyridoxal-5′-phosphate; GOL, glycerol.

### Cofactor Binding Site

**Figure 3:**
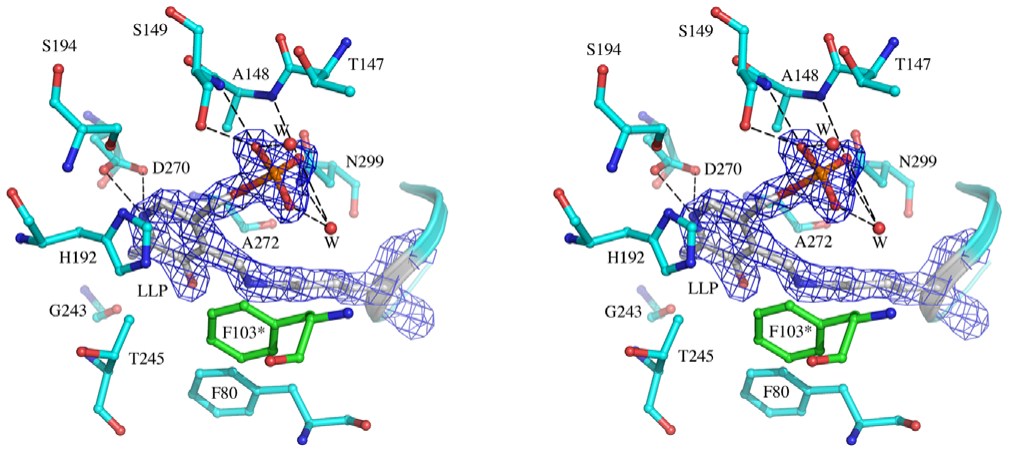
The drDDC active site. A stereo view of the active site in the drDDC structure. The LLP and the protein residues within a 4 Å distance of the cofactor are shown. Only the 2*F*_o_ - *F*_c_ electron density map covering the LLP is shown contoured at the 1.8 sigma. Hydrogen bonds are shown in dashed lines.

**Figure 4:**
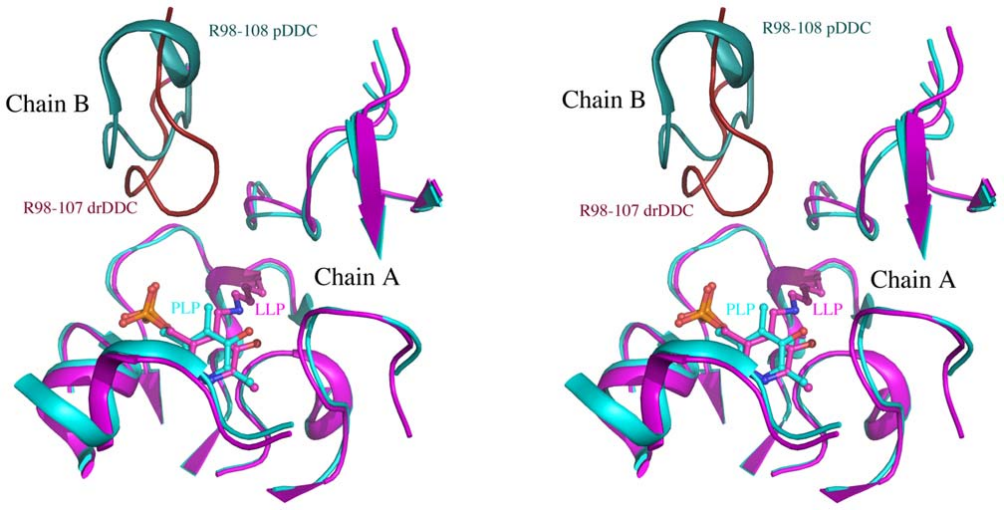
Superposition of drDDC structure onto pig DDC structure. The protein portions within 12 Å of the active center are shown in the schematic representation in stereo. Pig DDC and drDDC chain As are colored in cyan and magenta, respectively; and their chain Bs are colored in deep teal and brown, respectively.

The active site of drDDC is located near the monomer-monomer interface but is composed mainly of residues from one monomer (Fig. 3). The cofactor PLP binds to K302 through a Schiff base linkage to form an internal aldimine, lysine-pyridoxal-5-phosphate (LLP). The carboxyl group of D270 and the protonated pyridine nitrogen of PLP form a pair of salt bridges, which is structurally and functionally conserved within fold-type I of the PLP-dependent enzyme family. The cofactor is further anchored to the protein through an extended hydrogen bond network and hydrophobic interaction. The PLP pyridine ring is stacked between residues A272 on one side and H192 on the other side. The phosphate moiety of PLP is anchored by polar interactions with the peptide amide groups of residues A148 and S149 as well as by the side chains of S149, N299, and two water molecules. PLP also interacts with one residue from the other subunit, F103*. All residues involved in PLP binding in drDDC are conserved with those involved in the PLP binding sites in pig DDC. Upon superposing the native drDDC structure onto the pig DDC structure (Fig. 4), we identified a flexible loop in drDDC structure, which did not superpose well. This loop consists of residues 98–107 and is closer to the active center than the counterpart fragment, residues 98–107, of pig DDC. If drDDC binds a substrate, the loop (residues 98–107) must move away from the active site. Otherwise, there is not enough room for a substrate binding in the active center.

### Substrate Specificity and Kinetic Properties

Before testing the substrate specificity, we needed to identify a concentration of PLP that would not be rate-limiting for these reactions. We tested four concentrations, 0, 20, 40, and 80 µM of PLP, for each of the four recombinant proteins. Each enzyme showed similar activity at 20, 40, and 80 µM PLP in the reaction mixtures and about the half activity without PLP; thus using 40 µM PLP is sufficient for the enzyme activity assay. Because all the four recombinant proteins showed activity without adding exogenous PLP, they are likely to be holoenzymes. After two hours dialysis against 10 mM potassium phosphate buffer at pH 7.0, the enzymes still showed about the same activity when tested without adding exogenous PLP in the reaction mixtures, which suggests the enzymes are not apoenzymes; therefore we were unable to test enzyme K_M_ values towards PLP. During the expression and purification process, PLP became associated with some or most of the recombinant enzymes by forming an internal aldimine. Addition of exogenous PLP to the enzyme assay mixture increased the enzyme activity, which suggests that the enzyme had not been saturated with PLP; therefore, addition of PLP to the reaction mixture was necessary to achieve maximum activity for the enzyme.

**Figure 5:**
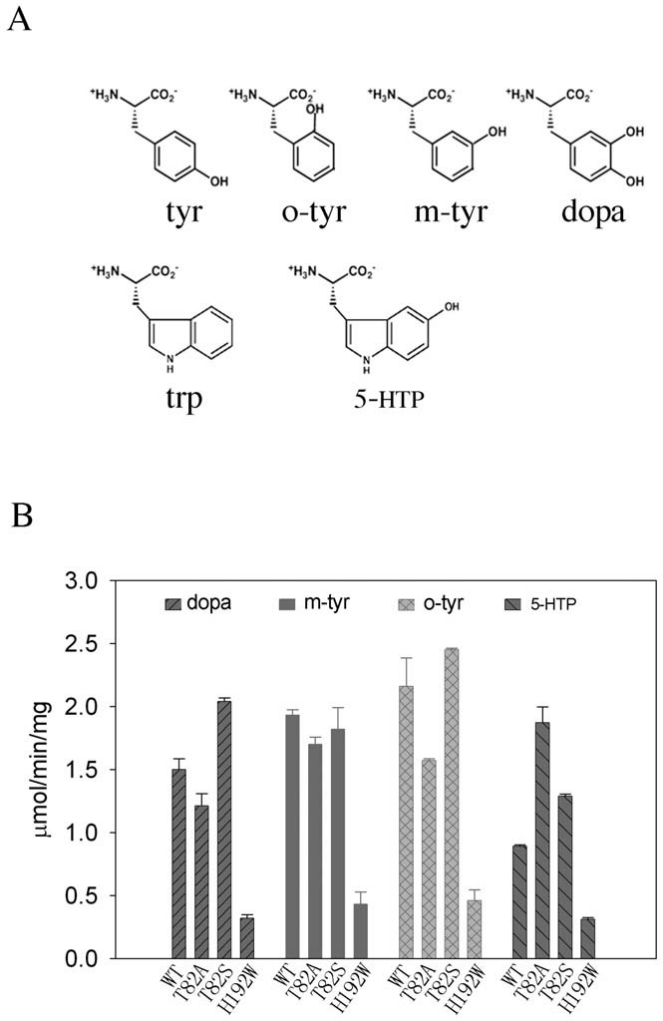
Molecular structures of tested chemicals and decarboxylation activity of drDDC WT and three mutants towards different aromatic amino acid substrates. *A,* Molecular structures of tyr (tyrosine), o-tyr, m-tyr, DOPA, trp (tryptophan) and 5-HTP; *B,* Decarboxylation activity. A typical reaction mixture of 100 µl consists of 5 µg of purified drDDC WT or any mutants, 1 mM of DOPA, 5-HTP, m-tyr, o-tyr, trp, tyr or d-DOPA, and 40 mM PLP in 150 mM potassium phosphate buffer, pH 7.0. Vertical axis shows the specific activity of the enzymes to each substrate (trp, tyr, and d-DOPA are not shown because none of the proteins showed any activity to them), and horizontal axis shows the drDDC WT and mutant protein names.

Substrate screening revealed that drDDC WT was active to DOPA and 5-HTP, but had no activity to tyrosine, D-DOPA or tryptophan. In contrast to mammalian DDC proteins that have higher catalytic efficiency to DOPA than to 5-HTP [34], drDDC has much higher affinity (5 times) to 5-HTP than to DOPA and also has greater catalytic efficiency to 5-HTP than to DOPA (Table 2). When DL-*o*-tyrosine (o-tyr) and DL-*m*-tyrosine (m-tyr) were used as substrates, production of *o*-tyramine and *m*- tyramine respectively were also observed (Fig. 5). Only the mixed DL forms of o-tyr and m-tyr are commercially available, but the inability of DDC to catalyze the decarboxylation of D-DOPA suggests that drDDC may be active to only the L-form of m-tyr and o-tyr.

**Table 2:** Kinetic parameters of drDDC WT and mutant proteins towards different substrates.

		K_M_ mM	*k*_cat_ min^−1^	*k*_cat_ /K_M_ min^−1^mM^−1^
Dopa				
	WT	2.2±0.3	282±1.6	128.2
	T82A	5.6±1	524±44.2	93.6
	T82S	4.5±0.9	672.6±61.9	149.5
	H192W	4.4±0.8	93.2±9.7	21.2
m-tyr				
	WT	2.3±1.2	213.8±40.4	93
	T82A	13.3±4.5	859.8±179	64.6
	T82S	11.4±2.2	690.5±80.2	60.6
	H192W	2.6±1.1	63.5±9.6	24.4
o-tyr				
	WT	1.1±0.3	110.8±8	100.7
	T82A	4.9±1.5	319.9±43	65.3
	T82S	1.9±0.7	225.7±28.2	118.8
	H192W	1.9±0.4	38.7±2.5	20.4
5-HTP				
	WT	0.4±0.04	62.8±1.4	157
	T82A	3.5±0.5	440.4±23.4	125.8
	T82S	0.6±0.1	102.6±3	171
	H192W	1.1±0.1	32.4±0.8	29.4

The activities were measured as described in Materials and Methods. The K_M_ and *k*_cat_ for different substrates were derived by using varying concentrations (0.1 to 12 mM)) of individual substrates. The parameters were calculated by fitting the Michaelis–Menten equation to the experimental data using the enzyme kinetics module. Results are means ± SE.

The crystal structure of pigDDC revealed that residues T82 and H192 are involved in substrate binding [33]. Structure alignment of drDDC and pigDDC demonstrates that drDDC has the same residues, T82 and H192, in these two positions. So we did a site-directed mutagenesis study for these two residues to confirm their roles in substrate binding. Because H192 is implicated in binding PLP as well, our study could also serve to substantiate its role in cofactor binding. Biochemical characterization shows all three mutants, like drDDC WT, show some activity to DOPA, 5-HTP, m-tyr and o-tyr (Fig. 5), but not to D-DOPA, tyrosine or tryptophan. However, the specific activity of H192W to all four substrates was significantly decreased; the specific activity of T82A mutant to DOPA and o-tyr was decreased and to 5-HTP was increased; and the specific activity of T82S to DOPA and 5-HTP was increased (Fig. 5).

Most significantly, all mutants decreased the ligand binding affinity as evidenced by their increased K_M_ values to all tested substrates. In particular, the K_M_ of the T82A mutant to DOPA was more than two fold higher, to m-tyr more than 5 fold higher, to o-tyr more than 4 fold higher, and to 5-HTP more than 8 fold higher; the K_M_ of the T82S mutant to DOPA was more than two fold higher, to m-tyr more than 4 fold higher; and the K_M_ of the H192W mutant to DOPA was more than two fold higher than that of drDDC WT to the same substrate. (Table 2) It was apparent that mutating T82 to alanine or serine significantly increased the enzyme turnover numbers (*k*_cat_) to all the substrates, from two to seven times, while mutation of H192 to tryptophan decreased the enzyme turnover number from two to three times. (Table 2)

## Discussion

Although there have been numerous studies dealing with insect DDC, particularly drDDC [2], the structural and biochemical properties of the enzyme have not been thoroughly analyzed in any insect species. Insects have more DDC-like sequences than mammals, which have only one DDC. For example, in *D. melanogaster*, *Tribolium castaneum, Anopheles gambiae*, and *Aedes aegypti* genomes, there are at least 5, 6, 7 and 7 DDC-like sequences respectively. In view of the presence of multiple DDC-like sequences in insects, a comprehensive understanding of the typical insect DDC is particularly important, because it is the basis for the classification of the other DDC-like sequences. We anticipate that a thorough knowledge of the typical DDC will provide important insights into the structure-function relationships of the other DDC-like sequences. Although partially purified drDDC has been documented as showing decarboxylation activity to tyrosine [35], we did not detect any decarboxylation activity to tyrosine using our purified recombinant drDDC. This finding, that drDDC has high activity to DOPA and 5-HTP and no activity to tyrosine, is consistent with a previous genetic study showing that drDDC is responsible for dopamine and serotonin production in *Drosophila*, but has no role in octopamine synthesis [36].

In contrast to pig DDC which shows higher affinity to DOPA than to 5-HTP [34], our data showed that drDDC had the highest affinity and catalytic efficiency to 5-HTP, although its turnover number for 5-HTP is lower than that for DOPA. Differences in the levels of DOPA or dopamine versus 5-HTP or serotonin in the brains of various species may be related to the differences in behavior among the various DDCs in terms of substrate specificity to 5-HTP and DOPA, possibly by requiring an enzyme to have a preferential affinity to compensate for lower levels of a particular substrate or product. For example, where the *Drosophila* brain has much higher levels of dopamine (600 pg/head) than serotonin (170 pg/head) [37], drDDC may be compensating with its greater affinity for the serotonin precursor, 5-HTP. However, in mouse brain, although the dopamine level is much higher than serotonin (135 vs 5 pg/µg protein) in the striatum, dopamine levels are lower than serotonin (0.7 vs 3.9 pg/µg protein) in the frontal cortex [38], where the serotonergic and dopaminergic neurons co-exist. Mammalian DDC may be compensating for these levels with a greater affinity for DOPA.

**Figure 6:**
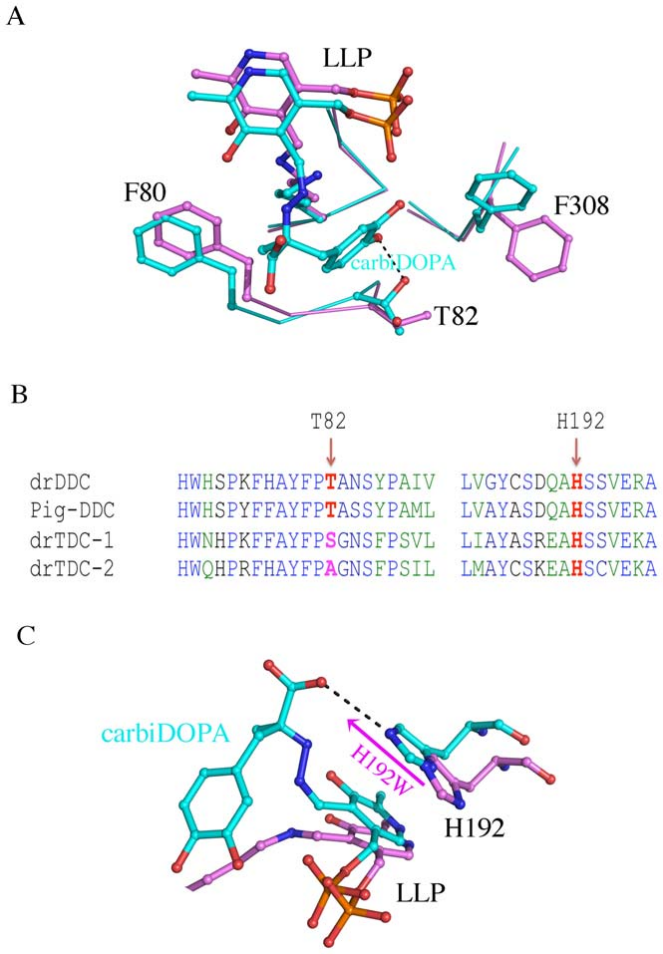
The roles of T82 and H192. *A,* Superposition of drDDC structure onto pig DDC structure shows that T82 of drDDC is a putative substrate binding residue. Residues from pig DDC are colored in cyan and those from drDDC are colored in magenta. *B,* Alignment of drDDC, pig DDC, *Drosophila* tyrosine decarboxylase-1 (drTDC-1) and *Drosophila* tyrosine decarboxylase-2 (drTDC-2). The corresponding residues of T82 and H192 are labeled. *C,* Superposition of drDDC structure onto pig DDC structure shows the H192 of drDDC is a putative substrate and cofactor binding residue. Residues from pig DDC are colored in cyan and those from drDDC are colored in magenta.

Although we determined the crystal structure of drDDC with the cofactor, PLP, at high resolution, we did not get a complex structure binding a substrate. The inability to obtain a crystal structure of substrate-bound enzyme might be a result of substrate turn-over by the enzymatic catalysis. To fully understand the mechanism of catalysis and substrate specificity, it will be necessary to co-crystallize the proteins (wild type and mutant drDDC) with an inhibitor or to perform molecular docking with consideration of protein conformational change. However, by superposing drDDC structure onto a pig DDC complex structure bound with the anti-Parkinson drug carbiDOPA [33], we were able to identify putative substrate binding residues of drDDC. Based on the information in the pig DDC-carbiDOPA complex, where the T82 residue forms a hydrogen bond with 4-catechol hydroxyl group of carbiDOPA (Fig. 6a), the corresponding threonine residue in drDDC is suspected to interact with a substrate. To provide more evidence of the role of T82 we did a site-directed mutagenesis study to see if altering T82 could affect the substrate binding. Usually, the dissociation constant is used to describe the affinity between a ligand and a protein. In the present study, we did not determine the dissociate constant of protein-ligand binding, but K_M_ values which are also indicative of the binding affinity between a substrate and an enzyme, with a lower value suggesting a higher binding affinity. The biochemical change of the mutants with replacement at T82 was smaller than expected. However, the K_M_ values of the mutants are changed by more than two fold for DOPA, which demonstrates that the binding affinity is changed by site-directed mutagenesis of T82. These results confirmed that T82 was involved in the substrate binding. But, it is not the residue determining the enzyme substrate affinity for DOPA as opposed to tyrosine. Structural alignment of drDDC, pig DDC, *Drosophila* tyrosine decarboxylase-1 and *Drosophila* tyrosine decarboxylase-2 shows that at the position of T82, both drDDC and pig DDC have a threonine residue, *Drosophila* tyrosine decarboxylase-1 has a serine residue and *Drosophila* tyrosine decarboxylase-2 has an alanine residue. (Fig. 6b) It appeared that T82 might be a critical residue to recognize DOPA in drDDC and that the alanine or serine at a similar position might recognize tyrosine. However, as with drDDC WT, the T82A and T82S mutants had no activity to tyrosine, suggesting that other critical residues are responsible for tyrosine binding. Furthermore, the pig DDC/carbiDOPA complex suggests that T82 may not be the critical residue which specifically recognizes DOPA, instead of tyrosine because T82 interacts with the *p-*hydroxyl group of carbiDOPA, which is present in both dopa and tyrosine. In the pig DDC/carbiDOPA complex, the NE atom of His302 forms a hydrogen bond (3.8 Å) with the 3-catechol hydroxyl group of carbiDOPA, and is only 4.2 Å away from the 4-catechol hydroxyl group of carbiDOPA [33]. The corresponding residue of His302 of pig DDC is His301 in drDDC, and Asn in two *Drosophila* tyrosine decarboxylases. It is possible that His301 in drDDC might be involved in DOPA recognition and the corresponding Asn residue in *Drosophila* tyrosine decarboxylases might be responsible for the recognition of tyrosine. Therefore, further sited-mutagenesis studies of His301 might provide further insight into the substrate specificity of drDDC and *Drosophila* tyrosine decarboxylases. Because the current structure is not a substrate-bound complex and a fragment near the active center is missing, further studies will be necessary to fully understand the substrate selection/recognition mechanisms of drDDC.

The crystal structure of drDDC shows that H192 interacts with the co-factor PLP pyridine ring. There is evidence that H192 may also interact with the enzyme substrate, as the corresponding histidine residue in pig DDC forms a hydrogen bond with a substrate like inhibitor (Fig. 6c). The alignment of drDDC, pig DDC, *Drosophila* tyrosine decarboxylase-1 and *Drosophila* tyrosine decarboxylase-2 shows that this residue is strictly conserved in all of these enzymes (Fig. 6b). Site directed mutation substituting tryptophan for the histidine residue, H192W, significantly decreased the activity, as well as the affinity evidenced by K_M_ change, to all the tested substrates. Although the tryptophan residue in the mutant should interact with the PLP pyridine ring in a manner similar to histidine residue of the wildtype, its larger size may also occupy the space for substrate binding, rendering it unable to form a hydrogen bond with a substrate. Compared to T82 mutations, the H192W mutation caused changes in the kinetic properties of drDDC, especially regarding the *k_cat_* value (Table 2). The PLP pyridine ring is stacked between H192 and A272; therefore, the H192W mutation may cause changes in the torsion between the pyridine ring and Lys-derived imine bond. This could affect the turnover number as well.

In summary, a specific DDC enzyme from fruit flies, drDDC has been functionally expressed, and its substrate specificity, optimal pH, temperature and crystal structure have been determined. A further site-directed mutagenesis study provides a reasonable biochemical and structural basis to propose T82 is involved in substrate binding and H192 is used not only for substrate interaction, but also for co-factor PLP binding of drDDC.

## Materials and Methods

### Chemicals

DOPA, D-DOPA, dopamine, L-tryptophan, 5-HTP, L-5-hydroxytryptamine, tyrosine, o-tyr, m-tyr, PLP and others were purchased from Sigma-Aldrich (St. Louis, MO, USA). All amino acids used in the experiment are L-form unless otherwise specified.

### First-Stand *Drosophila* cDNA Synthesis and Amplification of drDDC WT

Total RNA was isolated from *Drosophila melanogaster* larvae and pupae using TRIZOL reagent (Invitrogen, Carlsbad, CA) based on manufacturer's instruction and used to synthesize first-stranded cDNA. Oligonucleotide primers (Table 3) were synthesized and used for amplification of drDDC (NP_724164) from the synthesized fly cDNA pool.

**Table 3:** Oligonucleotide primers used for drDDC amplification and site-directed mutagenesis.

		Primer Sequences
WT	Fw	5′-AAAACATATGGAGGCGCCGGAGTTCAA-3′
	Rv	5′-AAAAGAATTCACTGCTCCTGTTCCATCTCG-3′
T82A	Fw	5′-AAGTTTCATGCCTACTTCCCCGCAGCCAACTCGTATCCAGCGATC-3′
	Rv	5′-GATCGCTGGATACGAGTTGGCTGCGGGGAAGTAGGCATGAAACTT-3′
T82S	Fw	5′-AAGTTTCATGCCTACTTCCCCAGTGCCAACTCGTATCCAGCGATC-3′
	Rv	5′-GATCGCTGGATACGAGTTGGCACTGGGGAAGTAGGCATGAAACTT-3′
H192W	Fw	5′-TACTGCTCGGACCAGGCTTGGTCATCCGTGGAGCGGGCTGGTCTT-3′
	Rv	5′-AAGACCAGCCCGCTCCACGGATGACCAAGCCTGGTCCGAGCAGTA-3′

### Expression and Purification of drDDC WT and Mutants

The amplified cDNA product for WT drDDC was cloned into an Impact™-CN plasmid (New England Biolabs) for expression of a fusion protein containing a chitin-binding domain. The frame and orientation of the DDC insert were verified by sequencing. T82A, T82S and H192W mutant recombinant DDC plasmids were prepared using recombinant drDDC Impact™-CN plasmid as a template by a QuikChange kit (Stratagene, USA) using the oligonucleotide primers listed in Table 3. *Escherichia coli* DE3 competent cells were transformed with the recombinant plasmids (containing WT or a mutant DDC sequence). Transformed bacterial cells were cultured at 37°C. After induction with 0.2 mM IPTG, the cells were cultured at 15°C for 24 hrs. The soluble fusion proteins were applied to a column packed with chitin beads and subsequently hydrolyzed under reducing conditions. The affinity purification resulted in the isolation of each individual recombinant protein at about 80% purity. Further purifications of the recombinant proteins were achieved by DEAE-Sepharose, Mono-Q and gel-filtration chromatographies. Purified recombinant DDC WT and T82A, T82S, H192W mutants were concentrated to 10 mg ml^−1^ protein in 10 mM phosphate buffer (pH 7), containing 40 µM PLP using a Centricon YM-50 concentrator (Millipore). Purity of the recombinant proteins was evaluated by SDS-PAGE and the concentration of the purified recombinant proteins was determined by a Bio-Rad protein assay kit (Hercules, CA) using bovine serum albumin as a standard.

### Effect of pH and Temperature on drDDC WT

To determine the effect of buffer pH on activity of drDDC WT protein, a 100 µL buffer mixture consisting of 100 mM phosphate and 100 mM boric acid was prepared and the pH of the buffer was adjusted to 6.0, 7.0, 8.0, 9.0, 10.0 and 11.0. 1 mM DOPA, 5 µg drDDC WT and 40 µM PLP were used in the reaction mixtures. To determine the effect of temperature, 100 µL of the same reaction mixture was used but with a different buffer (150 mM potassium phosphate buffer, pH 7.0). The mixture was incubated at temperatures ranging from 10–80°C for 10 min. During the mixture preparation for optimal temperature tests, drDDC WT protein was pre-incubated at the applied temperature for 1 min.

### Crystallization

The crystals were grown by hanging-drop vapor diffusion methods with the volume of reservoir solution at 500 µl and the drop volume at 2 µl, containing 1 µl of protein sample and 1 µl of reservoir solution. The optimized crystallization buffer consists of 15% PEG 4000, 20% glycerol, 200 mM CaCl2, and 100 mM Cacodylic acid, pH 6.5.

### Data Collection and Processing

Diffraction data of drDDC crystals were collected at the Brookhaven National Synchrotron Light Source beam line X29A (λ = 1.0908 Å). Data were collected using an ADSC Q315 CCD detector. All data were indexed and integrated using HKL -2000 software. Scaling and merging of diffraction data were performed using the program SCALEPACK [39]. The parameters of the crystals and data collection are listed in Table 2.

### Structure Determination

The structure of DDC was determined by the molecular replacement method using the published pig DDC structure (Protein Data Bank code, 1js3) [33]. The program Molrep [40] was employed to calculate both cross-rotation and translation of the model. The initial model was subjected to iterative cycles of crystallographic refinement with the Refmac 5.2 [41] and graphic sessions for model building using the program O [42]. The cofactor molecule was modeled when the *R* factor dropped to a value of around 0.24 at full resolution for the structures, based upon both the 2*F*o–*F*c and *F*o–*F*c electron density maps. Solvent molecules were automatically added and refined with ARP/wARP [43] together with Refmac 5.2.

### Structure Analysis

Superposition of structures was done using Lsqkab [44] in the CCP4 suite. Figures were generated using Pymol [45]. Protein and substrate interaction also was analyzed using Pymol [45].

### Substrate Screening

A typical reaction mixture of 100 µl consisted of 5 µg of purified WT or mutant drDDC, 1 mM of DOPA, tyrosine, L-tryptophan, 5-HTP, m-tyr, o-tyr or D-DOPA, and 40 µM PLP in 150 mM potassium phosphate buffer, pH 7.0. The reaction mixture was incubated for 10 min at 38°C and the reaction was stopped by adding an equal volume of 0.8 M formic acid into the reaction mixture. The mixture was centrifuged for 10 min at 15,000 g and supernatant (5 µl) was injected into a high-performance liquid chromatography reverse-phase column (150×4.6 mm, Varian, Palo Alto, CA) for analysis. The formation of decarboxylated products was monitored by an in-line UV detector. The formation of serotonin from 5-HTP and tryptomine from L-tryptophan was detected at a wavelength of 275 nm; dopamine from DOPA or D-dopa at 230 nm; tyramine from tyrosine, o-tyramine from o-tyr and m-tyramine from m-tyr at 225 nm.

### Kinetic Analysis

Once the activity of drDDC WT or a mutant protein with a tested substrate was verified, the kinetic parameters of drDDC WT and each mutant protein to the substrates were determined by incubating the protein in the presence of varying concentrations (0.1 to 12.0 mM) of the substrates at 38°C in a water bath incubator. The kinetic parameters were determined from the velocities of enzymatic reactions at different concentrations of substrates using the Michaelis–Menten equation by a non-linear regression method (Enzyme Kinetic Module in SigmaPlot 8, SYSTAT).

The atomic coordinate and structure factor (code: 3K40) have been deposited in the Protein Data Bank, Research Collaboratory for Structural Bioinformatics, Rutgers University, New Brunswick, NJ (http://www.rcsb.org).
